# Climate Change Mitigation and Adaptation in the Land Use Sector: From Complementarity to Synergy

**DOI:** 10.1007/s00267-014-0331-x

**Published:** 2014-07-22

**Authors:** Lalisa A. Duguma, Peter A. Minang, Meine van Noordwijk

**Affiliations:** 1 ASB Partnership for the Tropical Forest Margins and World Agroforestry Centre (ICRAF), United Nations Avenue, Gigiri, 30677, Nairobi, 00100 Kenya; 2 ASB Partnership for the Tropical Forest Margins and World Agroforestry Centre (ICRAF), 161, Jalan Cifor, Situgede, Bogor, 16001 Indonesia

**Keywords:** Adaptation, Complementarity, Land use, Mitigation, Synergy, Systems thinking

## Abstract

Currently, mitigation and adaptation measures are handled separately, due to differences in priorities for the measures and segregated planning and implementation policies at international and national levels. There is a growing argument that synergistic approaches to adaptation and mitigation could bring substantial benefits at multiple scales in the land use sector. Nonetheless, efforts to implement synergies between adaptation and mitigation measures are rare due to the weak conceptual framing of the approach and constraining policy issues. In this paper, we explore the attributes of synergy and the necessary enabling conditions and discuss, as an example, experience with the Ngitili system in Tanzania that serves both adaptation and mitigation functions. An in-depth look into the current practices suggests that more emphasis is laid on complementarity—i.e., mitigation projects providing adaptation co-benefits and vice versa rather than on synergy. Unlike complementarity, synergy should emphasize functionally sustainable landscape systems in which adaptation and mitigation are optimized as part of multiple functions. We argue that the current practice of seeking co-benefits (complementarity) is a necessary but insufficient step toward addressing synergy. Moving forward from complementarity will require a paradigm shift from current compartmentalization between mitigation and adaptation to systems thinking at landscape scale. However, enabling policy, institutional, and investment conditions need to be developed at global, national, and local levels to achieve synergistic goals.

## Introduction

**Fig. 1:**
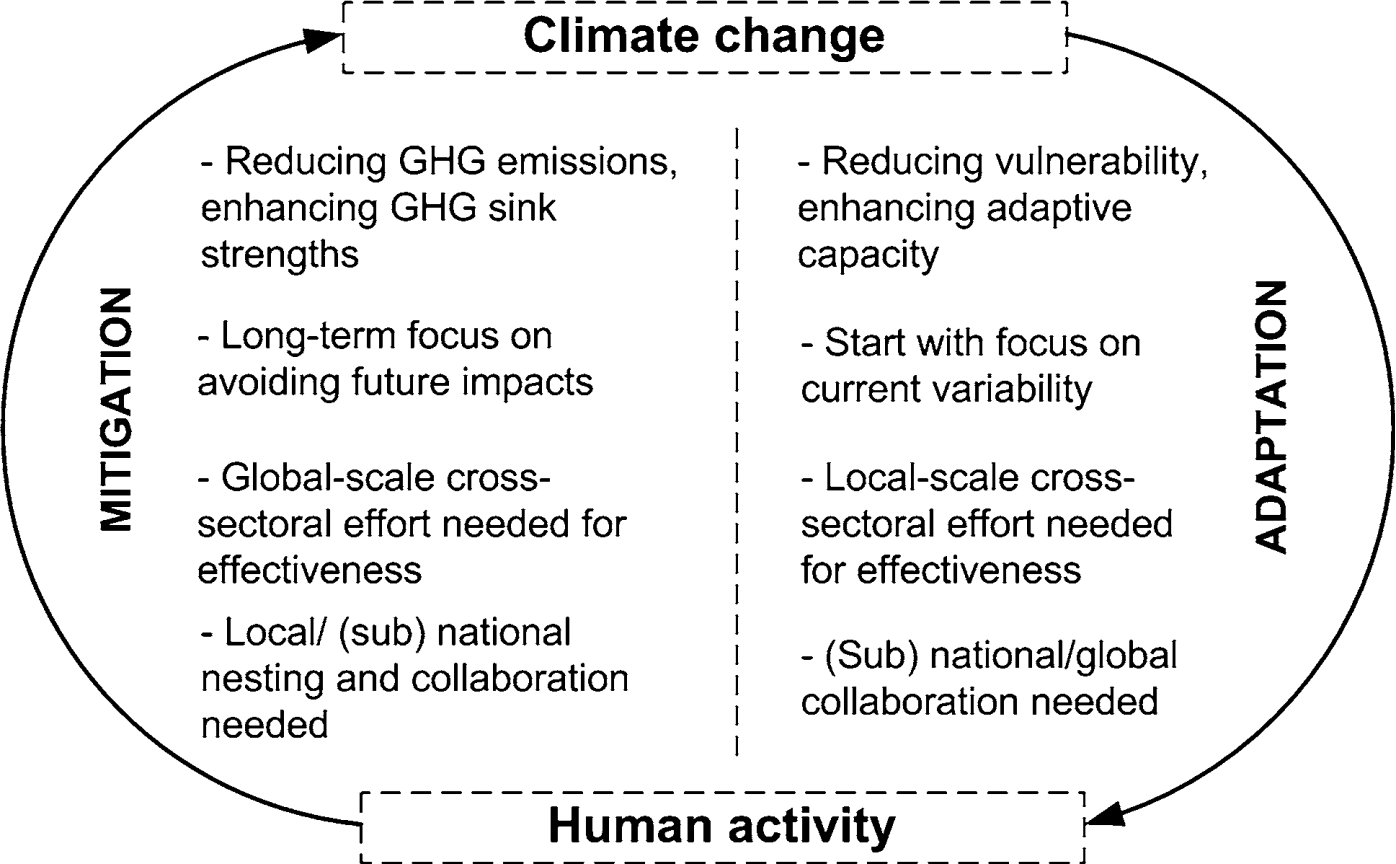
Climate change mitigation and adaptation as distinct interventions in the two-way relationship between human activity and global climate change. Note: the various comparative attributes are summarized from Dang et al. (2003), Tubiello et al. (2008) and Locatelli et al. (2010)

Mitigation and adaptation are the two primary instruments of the international climate convention to minimize negative impacts of climate change on humans and ecosystems. The less effective global mitigation is in reducing anthropogenic greenhouse gas (GHG) emissions and increasing GHG sinks, and the more adaptation is needed to avoid such negative impacts. Adaptation deals with enhancing the adaptive capacity and/or reducing vulnerability to climate change impacts while also taking advantage of the positive opportunities resulting from climate change. Despite both aiming to reduce the negative human and ecosystem impacts of climate change, the two measures are different in their specific objectives, scope, time dimension, and level of collaboration required (Wilbanks et al. 2003; Fig. 1).

The primary objective of the United Nations Framework Convention on Climate Change (UNFCCC), as stated in Article 2 is mitigation leading to “… *stabilization of GHG concentrations in the atmosphere* …..”, but within a time frame that allows “…*ecosystems to adapt naturally to climate change, to ensure that food production is not threatened and to enable economic development to proceed in a sustainable manner”*. In the first decade of the UNFCCC, there was hope that mitigation efforts would be adequate not requiring intensive active adaptation (van Noordwijk et al. 2011). With that hope gone, there still are distinct policy streams for mitigation and adaptation in current UNFCCC negotiations. Mitigation and adaptation are still implemented independently (Verchot et al. 2007; Locatelli et al. 2010), at different scales and are addressed by different groups of scholars (or institutions) each dealing with their own aspects of the two measures following different approaches (Ayers and Huq 2009). At the national level in developing countries, Nationally Appropriate Mitigation Actions (NAMA) are distinct from National Adaptation Programmes of Action (NAPA) and may be managed by different institutions. In Bangladesh, for example, adaptation is handled under the Ministry of Environment and Forestry, while mitigation is administered through a high-profile Designated National Authority (DNA) (Ayers and Huq 2009). All in all, the continued dichotomy has ‘carbonized’ the climate change discourse from the mitigation perspective to the detriment of salient issues of direct climatic effects of land cover (van Noordwijk et al. 2014) and land use that transcend the mitigation and adaptation divide. At national and subnational level, wherein implementation of climate change measures occurs, these dichotomies promote inefficiencies, unnecessary duplication, and most critically, contradictions in the minds of local farmers in developing countries who may not recognize these differences. Institutionally, the concept of ‘additionality’ that restricts mitigation finance to emission-reducing activities that would not otherwise happen can be in direct conflict with synergies.

Developing countries, which already have the lowest adaptive capacity and are bearing the heavy burdens of climate change impacts while they contribute little to GHG emissions deserve support for adaptation (UNFCCC 2001). At the Sixteenth Conference of the Parties (COP 16) in 2012, this need for international collaboration to assist developing countries to adapt to climate change impacts was formally acknowledged, although paling in comparison to the strong focus on mitigation in climate change dialogs over the last decades. Recently, arguments supporting the necessity for both adaptation and mitigation are growing (e.g., Laukkonen et al. 2009; Parry et al. 2001; IPCC 2001).

Despite the dichotomy at global and national levels and the differences in priorities for the two measures, Klein et al. (2007) stated that the opportunities for synergy between adaptation and mitigation measures are high in sectors like agriculture, forestry, and construction. However, so far, no specific work has been done to characterize the synergy approach and how it could be implemented. Little is known about what it takes to move from the current dichotomized approach to the synergy approach i.e., the necessary steps to be taken, the enabling conditions required, and the possible challenges that might be faced. To contribute to bridging these knowledge gaps, this paper aims to highlight key characteristics of synergy approaches and justify why the move toward such approaches is a necessary step in the land use sector. The drawbacks of the current climate policy were also examined and the necessary enabling conditions for synergy outlined. A case study from the Ngitili restoration system in Tanzania was used to illustrate some of our arguments backing the synergy approach.

## The Synergy Concept: A Theoretical Perspective

Corning (1995) stated that the concept of synergy exists in almost all forms of science even though the terminologies used to express it vary widely. Corning (1998) defined synergy as *“combined or ‘co*-*operative’ effects*—*literally, the effects produced by things that ‘operate together’ (parts, elements or individuals)”*. Classically, it has the context that “*effects produced by the wholes are different from what the parts can produce alone”* (Corning 1998). In synergy, two or more agents (von Eye et al. 1998), or components, or business units (Lazic and Heinzl 2011; Tanriverdi 2006) or interventions (in our case) are working together to achieve a jointly defined goal that matches all agendas. The main motive behind such an approach is increasing effectiveness, minimizing costs, and ensuring continuity of production and/or service provision by minimizing risks.

There are two major forms of synergy: additive and non-additive (von Eye et al. 1998). Additive synergy is the type of synergy where the desired effect or outcome is the sum of the independent effects of the agents or firms or interventions.

1 $$V\left( {x_{1} } \right) \, + V\left( {x_{2} } \right) \, + \ldots + V\left( {x_{n} } \right) \, = \, V\left( {x_{1} , \, x_{2, \ldots } x_{n} } \right) \, + \, I$$

where *x*_*1*_ *, x*_*2,…*_ *x*_*n*_ represent interventions/practices, *V* stands for the values/outcomes, and *I* is an interaction term being zero for the additive synergy case.

The second type, the non-additive synergy, is of three main categories: superadditive (*I* > 0 in Eq. 1), subadditive (*I* < 0 in Eq. 1), and isolated synergies (*I* depends on the specific set of *x*’s considered in Eq. 1). In superadditive synergy, the underlying principle is the concept of *the whole is greater than the sum of the parts* as there is an enhanced outcome when the components interact with each other (Corning 1998; von Eye et al. 1998). In the subadditive synergy model, the aggregate outcome when the interventions act together is less than the sum of the individual interventions outcomes. As a result, often some scholars view, this synergy model from its cost reduction value as that is also another goal of synergy. If costs are separated from benefits, the best result is obtained (Tanriverdi 2006; Tanriverdi and Venkatraman 2004) for superadditive *value* with subadditive *costs*. The third type of the non-additive synergy, the isolated synergy, is where the interaction between the interventions is the main focus irrespective of their individual effects (von Eye et al. 1998), for example, in chemical reactions.

Of the various forms of synergy, the most familiar one is the superadditive model and is even referred to as the classical conceptual model of synergy (von Eye et al. 1998). In this paper, we emphasize only the superadditive synergy model, as an approach to increase efficiency in addressing climate change (effectiveness per unit cost). In-depth empirical analysis relating to the models above is subjects of continuing research though we believe the Ngitili case study illustrated in this paper could suffice for the current context.

## Current Practices in Climate Actions: Complementarity

According to Klein et al. (2007), four major aspects of integration of climate change measures can be identified forming a potential platform for the synergy approach.

1. Mitigation actions with adaptation benefits.
2. Adaptation actions with mitigation benefits.
3. Processes that promote both mitigation and adaptation measures.
4. Policies and strategies that promote integrated mitigation and adaptation measures.

However, practices to-date largely emphasized the first two with limited attention to the last two despite them being necessary to progress along the synergy continuum. Table 1 shows some climate change related actions which largely emphasize the co-benefit context i.e., mitigation practices with adaptation benefits and vice versa, except the waste management in Bangladesh and the Ngitili system in Tanzania. There is limited emphasis on (1) the interactions and interconnections between the different practices and the associated processes and; (2) the policy and institutional integration aspects of synergy. Though the co-benefit provision is the very early and a necessary step toward synergy, synergy goes further in that it considers whether the co-benefits provided address the priority problems of the particular area, and whether the system-wide impacts of the co-benefits provision are positive and significant.

**Table 1:** Some differences between synergy and complementarity approaches to adaptation and mitigation measures in agricultural landscapes

	The synergy approach	The complementarity approach
Goal	Reducing impacts of climate change by addressing adaptation and mitigation within an integrated framework without prioritizing among the two and giving due attention to system integrity and functionality.	Reducing impacts of climate change by addressing adaptation and mitigation in such a way that either of the two is used as an entry measure providing the other one as a co-benefit.
Approach	The *whole* is more important than the *parts* and hence emphasizing more on integrated approach.	The *parts* are the priority and thus emphasis is given to the individual interventions.
Designing	Multi-stakeholders should be involved in order to ensure components integrity and system functionality	Often top-down approach mainly involving climate change professionals, donor agencies and target communities
Example 1	Agroforestry, ecosystem-based adaptation, climate smart agriculture	A forest plantation established for sequestering carbon but still providing services like micro-climate amelioration and habitat for wild life.
Example 2	Land sharing through multifunctionality (van Noordwijk et al. 2012)	Land sparing (Lusiana et al. 2012)

In the complementarity context, the emphasis was largely on the major–minor notion wherein either adaptation or mitigation was used as an entry measure and the other a co-benefit. Yohe and Strzepek (2007) stated that adaptation and mitigation could be complementary, in essence, because both end up in addressing climate change. Mitigation, in several instances, was even considered as a means of adapting to climate change (Dang et al. 2003). In contrast, in synergy, there is no prioritization of interventions during implementation; rather, emphasizing the mix of interventions to optimally achieve simultaneous multiple benefits while maintaining and enhancing system functionality. As much as possible, the combinations of interventions should reduce the negative impacts (tradeoffs) that would have occurred, which had the interventions been implemented individually. Thus, besides the multiple benefits of the practices, understanding and taking into account, the associated tradeoffs are central to synergy. Various studies (e.g., Bryan 2013; Bryan and Crossman 2013; Raudsepp-Hearnea et al. 2010) addressing synergy/co-benefits and tradeoffs particularly in ecosystems services context could provide important insights for this. Table 2 illustrates the major differences between synergy and complementarity.

**Table 2:** Some exemplary projects that are making use of the early stages of the synergy approaches at project and landscape levels

Name of project	Implementation approach	Aspects addressed by the project	Source
Scolel Te’ [Mexico]	Tipper (2002) stated that rather than going for how much carbon is sequestered, the project took the approach of first addressing the land use activities that communities and individual farmers were seeking to implement	1. Mitigation: carbon sequestration	Tipper (2002)
		2. Adaptation	
		3. Income generation for the rural households	
		4. Fuelwood and construction wood supply for households	
		5. Soil erosion reduction	
		6. Soil fertility enhancement	
Más Café’s under the AdapCC project [Mexico]	Addressing adaptation in an *integrated approach* wherein maintaining and increasing forest cover, pest management, carbon sequestration, energy efficiency, secure coffee drying process are the integral activities	1. Adaptation	http://www.adapcc.org/download/Final-report_Adapcc_17032010.pdf ^a^
		2. Mitigation: through carbon sequestration	
		3. Improvement of soil fertility	
		4. Enhancement of water supply	
		5. Income generation for the rural households	
		6. Reduction of soil erosion	
		7. Enhancement of energy use efficiency	
CEPICAFE Project under the AdapCC project [Peru]	Addressing the multiple problems in the landscape (e.g., lack of diverse income sources, erosion and landslides, drought, frostiness, strong winds, etc.) through reforestation and carbon sequestration, and capacity building and implementation of integrated coffee management practices. The aim of the project was to support farmers to improve the quality of their products, promote development within the sustainability context, and hence reduce poverty	1. Adaptation	http://www.adapcc.org/download/Final-report_Adapcc_17032010.pdf
		2. Mitigation: carbon sequestration	
		3. Income generation for the rural households	
		4. Enhancement of water supply	
		5. Soil erosion and landslide reduction	
		6. Soil fertility enhancement	
Waste-to-compost project [Bangladesh]	Improve the environment by promoting waste recycling	1. Mitigation: reduction of methane emission from waste	Ayers and Huq (2009)
		2. Adaptation: production of fertilizers to enhance soil fertility from boosting crop production	
		3. Adaptation: income generation for the urban and suburban poor	
		4. Sustainable development- job creation and pollution reduction	
The Kenya Agriculture Carbon Project [Kenya]	Carbon sequestration through the adoption of sustainable agricultural land management practices	1. Mitigation: carbon sequestration	http://web.worldbank.org ^b^
		2. Increasing agricultural yield and productivity	
		3. Enhancing exposure of Kenyan farmers to carbon market and revenues	
		4. Generating additional income sources for farmers through payment for ecosystem services	
Humbo Assisted Natural Regeneration Project [Ethiopia]	Rehabilitation of degraded forest lands for ecosystem services provision and community livelihood improvement	1. Mitigation: enhancing GHG removals by sinks	http://cdm.unfccc.int/ ^c^
		2. Provision of income stream for communities through sustainable harvesting of forest resources	
		3. Maintenance of water supply to the community	
		4. Promotion of native vegetation and biodiversity conservation	
		5. Reduction of soil erosion and flooding	
The HASHI project [Tanzania]	Ecosystem restoration using enclosures (Ngitili) and agroforestry practices	1. Carbon sequestration—REDD + pilot projects are underway	Monela et al. (2005)
		2. Restoration of ecosystem services, e.g., fuelwood, livestock feed, hydrological services, etc	

Note: These projects did not explicitly start as synergistic approaches, but resulted in being illustrative of such approaches through design decisions made throughout the process

^a^Accessed 27 December 2012

^b^Accessed 22 December 2012

^c^Accessed 21 December 2012

## Limitations of the Complementarity Approach

As described above, the current conceptualization of synergy within climate policies and various projects has not gone beyond the co-benefit context (complementarity). However, doing so has its own drawbacks which point to the inefficiency of the approach and implying the need for approaches that are integrative, efficient, and effective.

- First, by definition, it implies tradeoffs. In complementarity, it is difficult to achieve optimal benefits of both mitigation and adaptation. It is driven by either adaptation or mitigation.
- Second, complementarity is less cost effective in general. As Kane and Yohe (2000) argued, the treatment of adaptation and mitigation as different policy options increases the cost of climate change. Among the reason for this is the low integration of practices that could have minimized resource requirements. The poor integration of practices so far could be a precursor for poor institutional linkages, which also influenced the policy integration and sustainable development in general at international and local levels as argued by Tompkins and Adger (2005).
- Third, competition for resources between mitigation and adaptation (Tol 2005) is inevitable in complementarity obliging developing countries to prioritize among the measures e.g., the strong emphasis on adaptation by developing countries.

## Complementarity as a First Step in Synergy Continuum

**Fig. 2:**
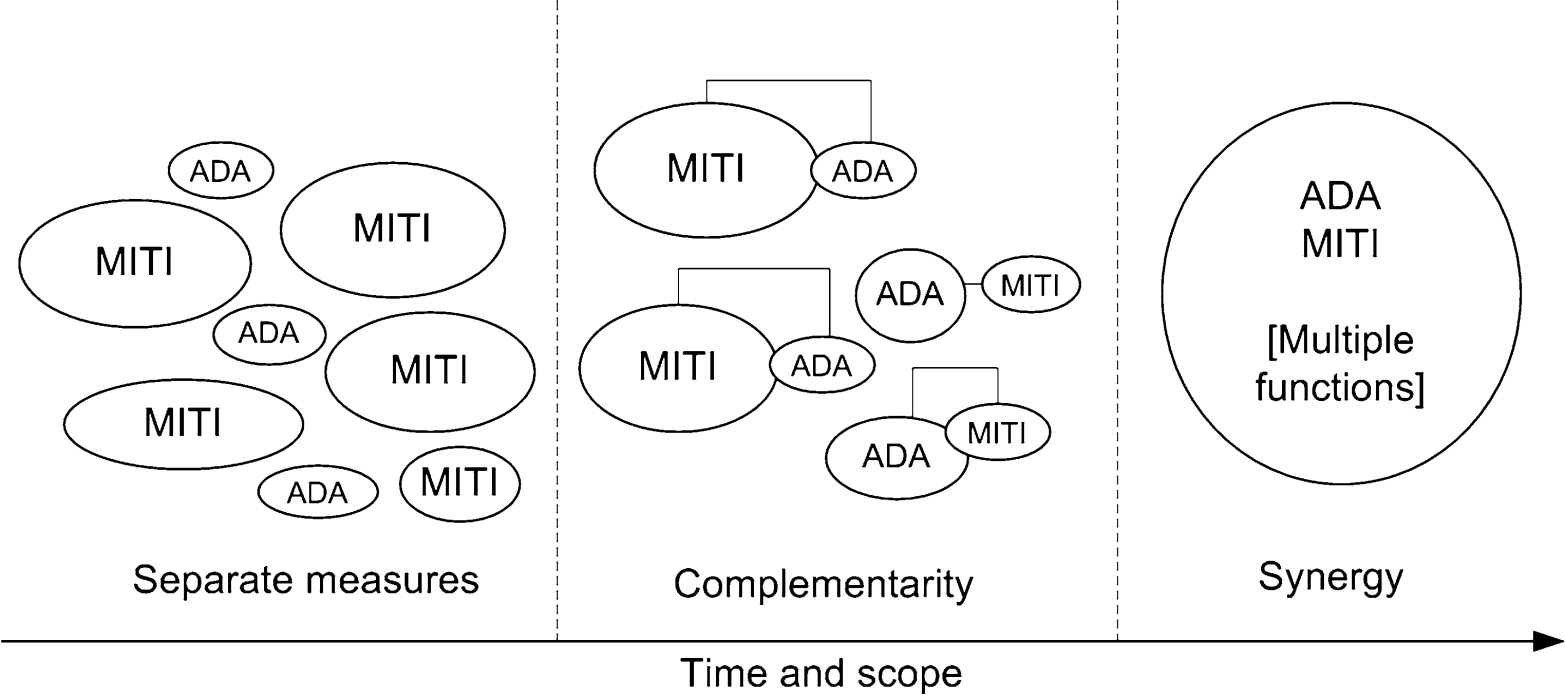
A schematic showing the complementarity and the synergy approaches to adaptation and mitigation measures. The size of the spheres is a relative indicator of the priorities for the measures with time. Note: MITI and ADA stand for Mitigation and Adaptation respectively

Figure 2 below illustrates the evolution of how adaptation and mitigation measures are addressed over time.

For synergy to happen, there should be resource relatedness (Lazic and Heinzl 2011; Tanriverdi 2006) and resource complementarity (Tanriverdi and Venkatraman 2004). Resource relatedness refers to a case where among two or more interventions, there exist resources to be shared, and there are similar activities between the interventions to be synergized. For example, in the land use sector, adaptation and mitigation share numerous resources: 1) land as a common necessity for both; 2) related practices e.g., afforestation, reforestation, agroforestry, silvopastoral systems; 3) skills of agriculture, forestry, and natural resource management and; 4) shared purpose—reducing the impacts of climate change. Resource complementarity on the other hand refers to a case when if the increase in one resource increases the return to the other resource (Milgrom and Roberts 1995; Harrison et al. 2001). There are a number of publications (e.g., Guariguata et al. 2007; Klein et al. 2007; Wilbanks et al. 2007) documenting that both adaptation and mitigation enhance the effectiveness of one another.

Klein et al. (2005) argued that adopting the synergy approach could enhance the cost-effectiveness of climate change measures. Two reasons underlie the efficiency and effectiveness associated with the synergy approach. The first is the fact that mitigation and adaptation capture two key components of climate policy. For instance, Tubiello et al. (2008) stated the integrative nature of synergy approach makes it the core of climate policy at multiple scales in the future. The second reason is the strong resource relatedness and resource complementarity between adaptation and mitigation measures in the land use sector. Klein et al. (2005) highlighted that if achieved, such efficiencies (resulting from the resource relatedness concept) could make the practices attractive to land users and to those engaged in making decisions about climate change measures. Such efficiency and effectiveness attributes of synergy also make it a potential approach for addressing issues of food, energy, and water supply.

The move from complementarity to synergy requires identifying the right approaches and concepts that enhance multifunctionality ensuring the provisions of simultaneous benefits. The landscape approach, which puts particular emphasis on multifunctionality and interactions among components, is very helpful in the move toward synergy. Another much related approach to the landscape one is the ecosystem services concept. For example, according to De Groot et al. (2010), contextually there is almost no distinction between ecosystem services and landscape functions. In the land use sector, most landscape functions can be expressed directly or indirectly by one or more ecosystem services. For example, according to MA (2005), climate regulation (e.g., carbon sequestration and effects of land cover on climate parameters) is a regulating ecosystem function that mainly contributes to mitigation potential, while the provisioning, regulating, habitat, and supporting ecosystems functions boost adaptation. It is thus arguable that ecosystem services could serve as a potential strategy for enhancing synergies between mitigation and adaptation.

## Moving from Complementarity to Synergy to Address Climate Change in the Land Use Sector

In our view, the move from complementarity to synergy particularly to achieve the superadditive value and subadditive cost models needs to capture four key elements:

1. Identifying the practices;
2. Understanding the processes;
3. Addressing tradeoffs and;
4. Formulating supportive policies.

The following sections deal with each of these elements in further details.

### A Portfolio of Practices and Their Interconnectedness

In agricultural landscapes, there are considerable linkages between mitigation and adaptation. A number of studies have highlighted this, for example, in agriculture by Harvey et al. (2013), Rahn et al. (2013) and Rosenzweig and Tubiello (2007) and in the forestry sector by Kane and Shogren (2000), Dang et al. (2003), Klein et al. (2005), Ravindranath (2007), and Wilbanks et al. (2007). Tompkins and Adger (2005) stated that there is a clear interdependence between adaptation and mitigation actions as they are driven by common factors such as the availability and penetration of new technologies and the capacity and readiness of the society for change. Practice portfolios in synergy should therefore enhance both adaptation and mitigation benefits while addressing other development and conservation needs. Some examples of such practices include agroforestry, soil conservation, ecosystem-based adaptation, and climate smart agriculture.

**Fig. 3:**
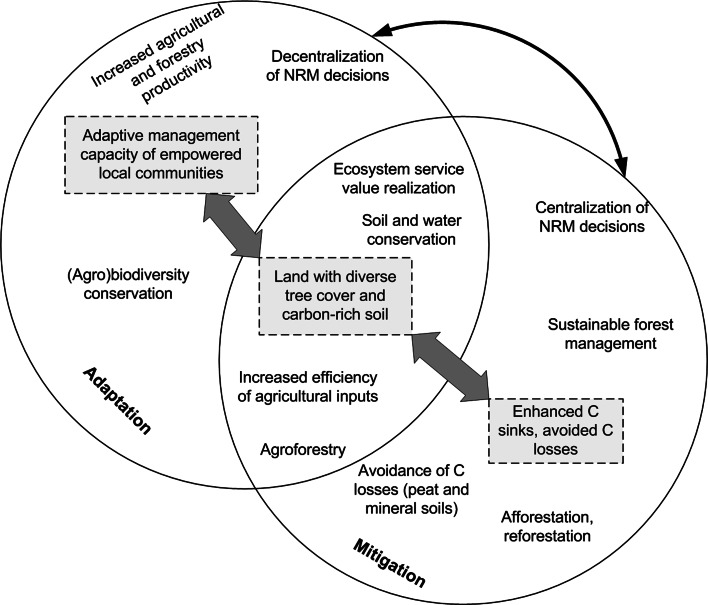
The interrelationships between adaptation and mitigation measures in rural landscapes. NRM denotes natural resources management and C stands for Carbon

In communities where livelihood is based on land resources, the success of mitigation measures depends on how good the community adapts to the prevailing conditions (e.g., drought, erratic rainfall, flood, etc.). For example, mitigation measures such as afforestation, reforestation, and sustainable forest management are very susceptible to community livelihood conditions, because driven by poverty people may illegally exploit forests thereby affecting carbon sinks hence mitigation efforts. The strong interconnectedness of the processes, decisions, and interventions (Fig. 3) challenges our conventional fragmented approaches to problems in the land use sector. Hence, for effective mitigation or adaptation actions, taking holistic approaches that consider community livelihood, natural resources management and other biophysical, policy, and institutional aspects are required. One way of moving toward such holistic approaches while capturing the above-mentioned diverse and strong interconnections, inter-linkages, and interdependencies is the systems thinking concept.

The adoption and implementation of systems thinking approach to climate change measures require proper policies, strategies, and institutions that favor the approach. For instance, if a state’s policy emphasizes only economic returns from land uses without considering their ecological and social implications, it is challenging to implement integrated system-wide approaches.

### Processes Necessary to Move towards the Synergy Approach

**Fig. 4:**
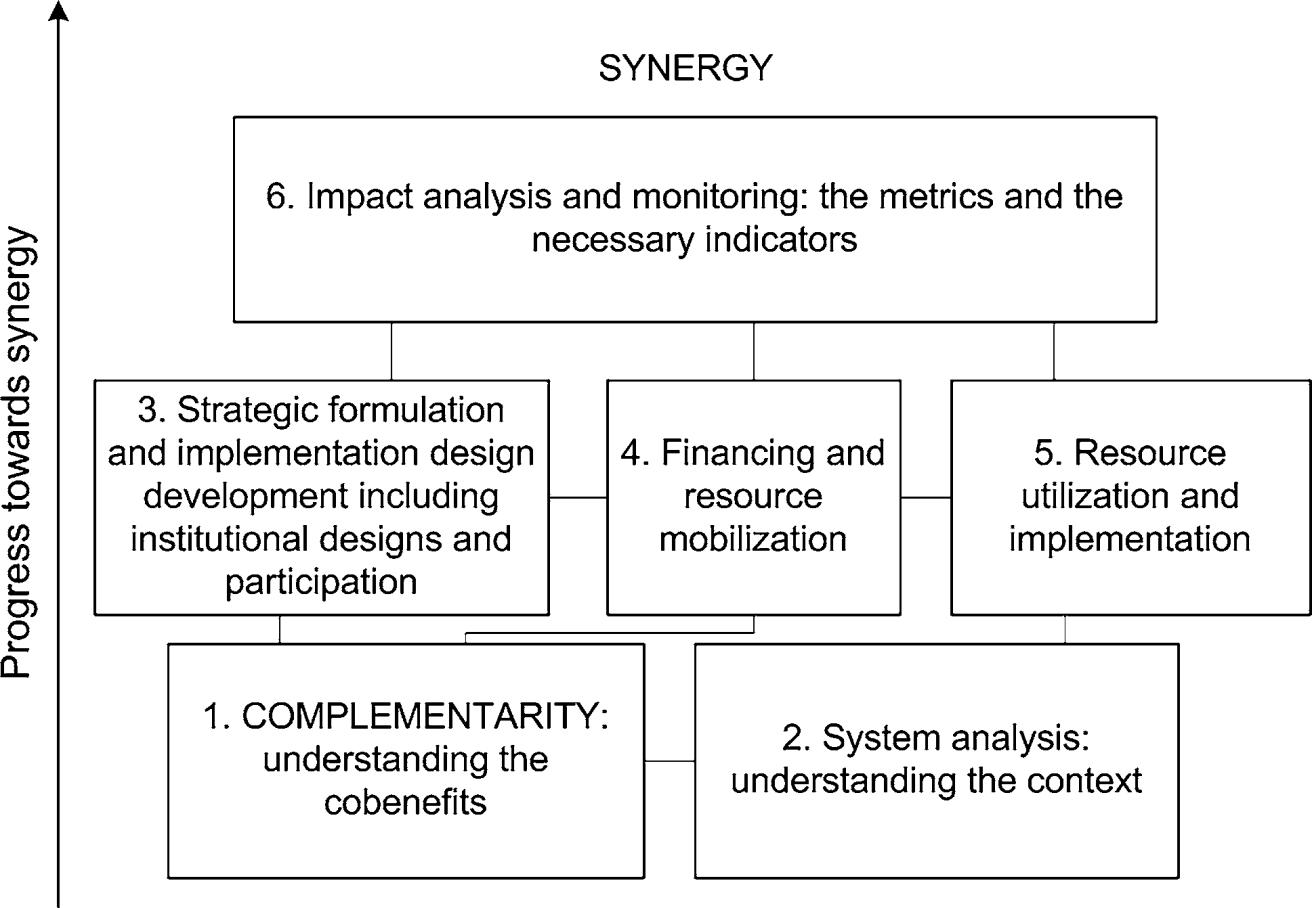
The hypothetical national or project level processes to move from complementarity to synergy between mitigation and adaptation

The synergy approach involves numerous processes. For the sake of simplicity, we synthesized some processes necessary for projects or programs that intend to employ the synergy approach to address climate change issues. However, depending on the local contexts of the projects/programs, other relevant processes may be added too. The first important step is to identify the extent of complementarity, because it is a prerequisite for synergy to happen (Fig. 4). This largely emphasizes exploring the multiple benefits from the mix of practices. The system analysis process (no. 2 in Fig. 4) is crucial in synergy and involves identifying the system components, how they function and interact and how good the selected measures fit into the local context. It intends to identify the tradeoffs associated with the practices and craft strategies for its possible reduction. Even in countries possessing an integrated climate policy, this process is often overlooked or simplified and sometimes is overshadowed by environmental impact assessment activities that rarely go beyond investment project perspectives.

Processes 3–5 in Fig. 4 designate the synergy planning phase which addresses the creation of the right institutions, defining the financing mechanism, and ensuring the involvement of the necessary stakeholders in the process, i.e., participatory approach. It embraces efforts toward cross-sectoral and interdisciplinary planning approaches whereby climate change is mainstreamed into sectoral polices thereby enhancing the integration process. Some examples include the Low Carbon Agriculture Programme of Brazil, the Climate Resilient Green Economy Strategy of Ethiopia and the National Action Plan on Climate Change of Indonesia. Processes 3–5 also form the basis for the creation of the necessary processes linking the national and subnational governments to the local level. Such processes may have binding agreements that define rules/procedures, rights, and responsibilities among the actors especially between the national and local ones.

To ensure multifunctional initiatives like synergy function properly, multiple long-term financing mechanisms (process no. 4 Fig. 4) are required as argued by Bryan and Crossman (2013) for ecosystem services provision. Such arrangements may reduce risks due to unforeseen circumstances (e.g., budget cuts) while possibly increasing the trust of the local communities for the initiatives. Process no. 6 (Fig. 4) focuses on designing and developing the required metrics, i.e., criteria and indicators to properly assess benefits and impacts of synergy.

### Addressing Tradeoffs between Mitigation and Adaptation Measures in the Land Use Sector

Identifying and dealing with tradeoffs is as important as exploring the potential for synergies between mitigation and adaptation though the former received little attention thus far. With closer scrutiny, a number of tradeoffs can be identified between mitigation and adaptation when the two are treated separately (Harvey et al. 2013). For example, the expansion of fast-growing tree species like Eucalyptus in the highlands of Ethiopia resulted in considerable carbon sequestration though the species was often blamed for intense water consumption due to its growing habit and thus constraining water availability for local communities. Kidanu et al. (2004) also observed the species competed with adjacent crops significantly thereby reducing crop productivity. Tree-crop biofuels expansion, a mitigation strategy to replace fossil fuels with renewable energies, has also faced backlashes in different parts of the world due to its competition for crop production areas despite sequestering considerable amount of carbon in the medium term and reducing the use of fossil fuels for energy in the long term. Bryan et al. (2010) illustrated that despite the first generation biofuels being attractive in their mitigation potential, they negatively influenced food production under different scenarios. Asquith et al. (2002) also argued that carbon projects that result in large-scale land use changes may influence community livelihood by limiting access to land and other resources besides their impact on biodiversity. Livestock (particularly in drought prone areas) is considered a common coping strategy to drought and famine despite contributing about 18 % of the anthropogenic GHGs emissions (Herrero et al. 2009). Rahn et al. (2013) found that promoting soil conservation practices and adequate fertilization of coffee agroforestry systems implied increased adaptation potentials while providing limited mitigation benefits.

Though identifying tradeoffs is important, strategies to minimize it are equally necessary. In crop production systems, practices such as conservation agriculture (Hobbs et al. 2008), agroforestry (Verchot et al. 2007), and soil and water conservation (Ravindranath 2007) could reduce the extent of possible tradeoffs. In the forestry sector, use of diverse tree species in plantations (Ravindranath 2007), growing native tree species (Ravindranath 2007), tree plantings in degraded and marginalized lands, adoption of sustainable forest management, and enclosure systems in dryland areas (Duguma et al. 2013) could be considered potential strategies to minimize tradeoffs. In the livestock sector, growing leguminous fodder trees and adopting silvopastoral systems could play crucial role in minimizing tradeoffs.

Harvey et al. (2013) argued that occurrence of tradeoffs varies by time and scale implying the need for time and scale sensitive strategies to address it. For instance, integrating nitrogen fixing trees into farms reduces land area of production; however, in the long run, such trees could enhance soil fertility and thus increase productivity.

### Policies for Promoting Synergies between Mitigation and Adaptation within the Multifunctionality Context

National and subnational policies and strategies are crucial for the implementation of multifunctional interventions which provide mitigation, adaptation, development, and conservation benefits simultaneously. Through this, synergies between mitigation and adaptation could be more practical and also engaging. Such policy related supports for synergy could be through: 1) creation of the right institutions; 2) establishment of long-term multiple financial mechanisms (e.g., arranging mechanisms of funding multifunctional projects through international donors supports); 3) developing and implementing policy incentives for either private or communal multifunctional projects, for example, through land tenure clarification; 4) empowering local communities and technical backstopping for committed engagement through extension schemes. Though this list is not comprehensive, it highlights that national and subnational governments can help the implementation of multifunctional processes through proper policy formulations. Such moves can be integral components during the designs of cross-sectoral policies and strategies at various scales.

Policies and strategies are also crucial in determining the practices and processes that might be adopted in interventions that promote multifunctionality. They can guide the nature of decisions that should be made besides defining who makes those decisions during the initiative’s design and implementation. These, in some cases, may include decisions on tradeoffs and who should have the power to make the choices (and the priorities) in order to minimize the tradeoffs. Policies may also guide the institutional arrangements required and the financing schemes necessary for multifunctional initiatives like synergy.

## An Illustrative Case Study: Applying the Non-Additive Synergy Model to the Ngitili System in Shinyanga, Tanzania

Shinyanga region is a semiarid area in Northern Tanzania receiving average annual rainfall of 700 mm. Its inhabitants are largely agropastoralists and the region hosts almost 20–30 % of the country’s livestock population. Its vegetation type is mainly extensive Acacia and Miombo woodlands (Monela et al. 2005).

**Fig. 5:**
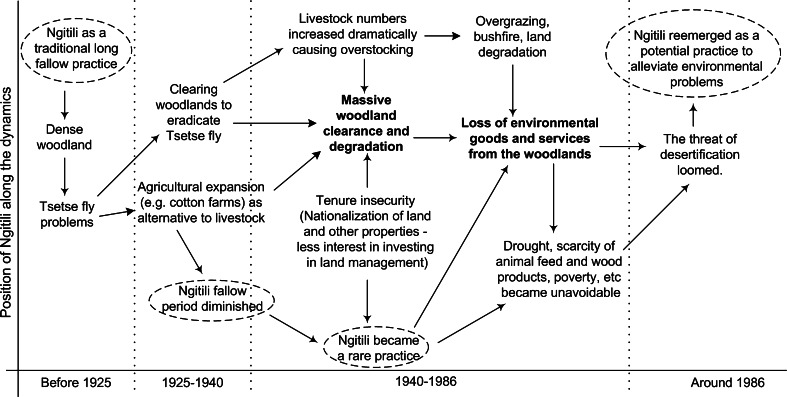
The position of the Ngitili along the dynamics in the Shinyanga region

Ngitili, a practice involving regeneration and conservation of trees on cropping and grazing lands, is a traditional fodder bank system used to conserve pasture for the dry season in Tanzania (Mlenge 2004). Due to a complex set of factors (Fig. 5), the practice was abandoned in 1920 s and was reintroduced in 1980 s after realizing its potential against the desertification problem that threatened the region. Figure 5 shows the problem context and the underlying processes in this transition. This strongly relates to the process no. 2 (system analysis*)* illustrated in Fig. 4.

Together with other practices such as rotational woodlots, improved fallows, and homestead planting, Ngitili was promoted in Shinyanga as a means to reduce poverty and promote livelihood security through ecosystem restoration efforts (Mlenge 2004; Monela et al. 2005). The Ngitili-based restoration had a strong national support (Mlenge 2004). Some key policy and strategic measures taken by the government to promote the restoration include 1) institutionalization of the region-wide program known as HASHI (Shinyanga Soil Conservation Programme) in the 1980 s supported financially by the Royal Norwegian government and technically by the World Agroforestry Centre (ICRAF); 2) creation of a funding mechanism such as the Shinyanga Mazingra Fund which supported grassroot initiatives and the channeling of the bilateral support from the Royal Norwegian government toward this program; 3) empowerment of the local institutions and adoption of the local practices to ensure the intense engagement of local communities; 4) creation of village environmental committees who had strong voice in the dialogs and decisions on matters relating to the program; 5) the enactment of the 1997 Land Policy and the Land and Village Land Acts of 1999 that enabled villages and its members to have land title deeds which supported the formal establishment of Ngitili (United Nations Development Programme (UNDP) 2012).

Such strong measures and policy supports from the national government (together with the intense engagement of the local communities in the program, the multiple financial mechanisms and the sustained technical support from ICRAF) propelled the restoration effort remarkably, i.e., from around 611 ha in 1986 (Mlenge 2004) to at least 350,000 ha by 2004 (UNDP 2012). Thus, to realize multifunctional initiatives like synergy, such supportive policies and multi-institutional engagements which value the voices of the locals are necessary.

**Fig. 6:**
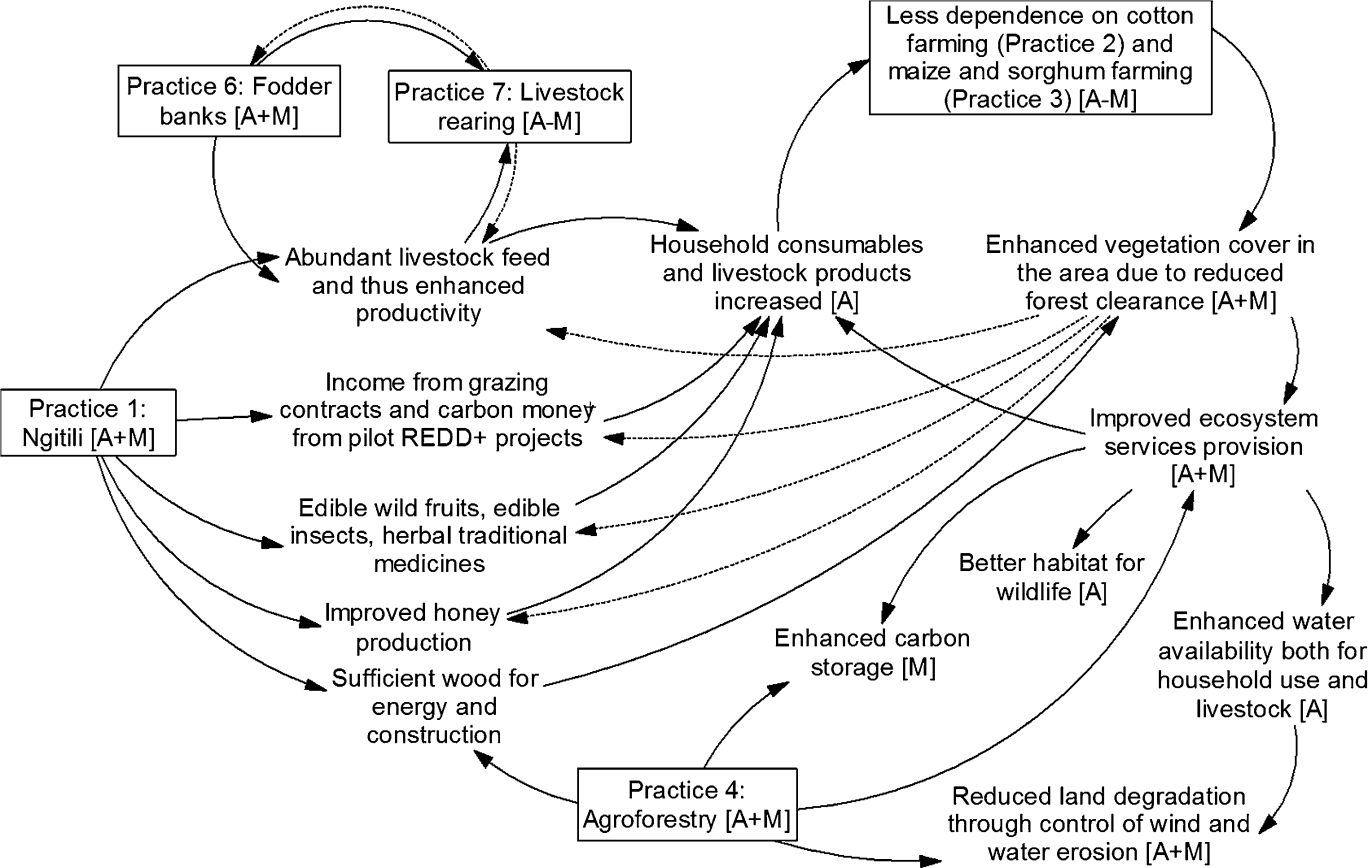
Practices and their interconnections in the Ngitili system in the Shinyanga region. Note: A- Adaptation; M- Mitigation; A + M denotes the practice contributes positively to both adaptation and mitigation; A − M denotes the practice positively contributes to adaptation but affects mitigation

The reintroduction of Ngitili played a major role in addressing climate change issues though the implementation was neither as adaptation nor as mitigation but rather as a multifunctional approach encompassing a pool of practices. Figure 6 shows the key practices in the landscapes where Ngitili restoration was taking place and how they interact and influence each other. Ngitili’s potential to provide simultaneous multiple functions makes it a good illustration for the superadditive synergy model. These functions could be expressed in one or more ecosystem services, hence supporting our earlier argument that ecosystem services could serve as a vehicle to promote synergies between mitigation and adaptation measures.

With average carbon (C) stock of 45 t/ha (Monela et al. 2005), the Ngitili system sequestered around 23 million t C by 2000 (Barrow and Shah 2011). Recognizing this sequestration potential, REDD + pilots have already commenced in the area. Ngitili expansion also generated additional benefits which boost the adaptive capacity of the community. For example, due to catchment conservation and other hydrological services of Ngitili vegetation, water availability for household use and for livestock is increasing and even there are small dams constructed by the community to accumulate water for the dry season (Mlenge 2004). The availability of edible items also increased after Ngitili restoration. The annual provisions of 534 liters of milk, 14 kg bush meat, 26 kg mushrooms, 33 liters of honey, and 30 kg of fruits were associated with Ngitili (Monela et al. 2005). Over 25 medicinal plants used to treat over 20 different diseases were also recorded in restored Ngitilis.

Ngitili expansion proved to be a strong economic boost for the whole region with an increase in value of around $23.7 million (Monela et al. 2005). The per capita per annum economic value of a restored Ngitili was around $168 (Barrow and Shah 2011), considerably surpassing the national average rural expenditure ($102). Ngitili also provides numerous social and environmental benefits (Monela et al. 2005): 1) conflicts on grazing areas and on woodland products collection reduced; 2) children can now attend schools as livestock can be kept around Ngitilis; 3) wood products became easily available e.g., fuelwood collection time reduced between 2 and 6 h for women (Barrow and Shah 2011); 4) a favorable habitat for wildlife was created, for instance, restored Ngitilis were home for up to 145 bird and 13 mammal species (Barrow and Shah 2011).

Despite the numerous functions of Ngitili restoration, there are a number of tradeoffs in the system. Some of them are: 1) livestock are among the important contributors to GHG emissions despite being also the livelihood basis of this community; 2) the increasing expansion of enclosure-based fodder management system competes with the land available for crop production; 3) there is a poor market access for the products though currently most products seem to be consumed locally; 4) most recent discussions emphasize the expansion of Ngitili with limited look at the long-term implications of the expansion, for example, possibilities of woodland invasion which in the long run can enhance carbon sequestration but may limit the livestock feed production.

## Concluding Remarks and the Way Forward

We set out to examine how the synergy concept is currently conceptualized and concluded that the current notion does not differ from complementarity. Complementarity, however, is not sufficient to address the existing and projected impacts of climate change especially in the land use sector. Thus, climate policy should start looking at the next possibility, synergy, which has often been overlooked or sometimes confused with complementarity.

The transition from complementarity to synergy requires shifting from the co-benefit context to systems approach that embodies a set of practices that provide simultaneous multiple functions. We suggest four crucial elements necessary in the move toward synergy: 1) identifying the practices and their interconnectedness; 2) examining the processes and their interrelationships; 3) addressing the tradeoffs; and 4) supportive policies and strategies. Understanding the practices and the processes and their interactions is the key to minimize the costs of climate policy. This in a way is by looking at the resource relatedness and resource complementarity between mitigation and adaptation. A closer look at the illustrated case study on Ngitili system showed that processes that link the national systems to the local practices through policies and strategies are necessary especially to ensure the necessary policy support, to put in place appropriate financing schemes, to remove obstacles for the implementation of multifunctional initiatives. For example, the move taken by the Tanzanian government in ensuring land tenure security to promote the Ngitili restoration is exemplary.

The financing scheme is crucial to implement multifunctional initiatives like synergy as the current mode of budget allocations at national, subnational, and local scales is often earmarked with specific practices which do not encompass the whole spectrum of synergy. Some financing schemes that hold potentials include the payment for ecosystem services (PES), the co-investment mechanisms (Namirembe et al. 2014), and the emerging REDD + scheme. However, identifying the right financing mechanism to effectively implement synergy remains an open area of research.

Besides the financing, below are some selected key challenges to the synergy approach.

- The dichotomy created at UNFCCC in treating mitigation and adaptation as separate policy measures;
- The strong emphasis of UNFCCC on achieving stabilization of GHGs (UNFCCC *Article 2*) and looking at the adaptation aspect as a mechanism to achieve the mitigation objective;
- The lack of proper metrics (criteria and indicators) for analyzing the benefits of synergy approach;
- The scientific uncertainty regarding the optimal mix of practices to achieve maximum benefits out of synergy (Klein et al. 2005; Dang et al. 2003).

For enhancing the efficiency and effectiveness of climate policy using synergy approach, 1) intense lobbying and dialogs with the concerned bodies are necessary to address the first two challenges; 2) research needs to focus on the last two challenges; 3) enabling policy, institutional and investment conditions for synergy need to be developed at global, national, and local levels. In sum, synergy is a continuum, which could be achieved by targeting the superadditive synergy model presented earlier.
